# *Dendrobium officinale*-derived nanovesicles: a natural therapy for comprehensive regulation of angiogenesis, inflammation, and tissue repair to enhance skin wound healing

**DOI:** 10.1186/s40643-025-00915-3

**Published:** 2025-07-12

**Authors:** Jin Tu, Luhua Xu, Yuqin Guo, Minzhi Zhang, Miao Gan, Xiuzhen Bao, Rongfeng Yang, Hanjiao Liu, Fengxia Lin

**Affiliations:** 1 Department of Urology, Shenzhen Hospital of Integrated Traditional Chinese and Western Medicine , Shenzhen, 518104 Guangdong China; 2 Department of Cardiovascular, Shenzhen Bao’an Traditional Chinese Medicine Hospital, Guangzhou University of Chinese Medicine, Shenzhen, 518100 Guangdong China; 3 Division of Cardiovascular Intensive Care (CICU), Cardiac and Vascular Center, The University of Hong Kong-Shenzhen Hospital, Shenzhen, 518053 Guangdong China; 4 The Seventh Clinical Medical College of Guangzhou University of Chinese Medicine, Shenzhen, 518100 Guangdong China

**Keywords:** Skin wound healing, Angiogenesis, Plant-derived nanovesicles, AKT/eNOS

## Abstract

Skin wound healing is a multifaceted biological process involving dynamic interactions among various cells and signaling molecules. Angiogenesis is a key component of this repair process. Dendrobium officinale, a traditional medicinal plant, has shown therapeutic promise, particularly through its bioactive nanovesicles. This study investigates the therapeutic potential of Dendrobium officinale-derived nanovesicles (DDNVs) in regulating angiogenesis, inflammation, and tissue repair, to promote enhanced skin wound healing. A full-thickness mouse skin wound model was used to evaluate the in vivo effects of DDNVs on wound closure, angiogenesis, and collagen remodeling. Histological staining (H&E and Masson’s trichrome) and CD31 immunofluorescence were performed. In vitro, DDNVs were tested on Human umbilical vein endothelial cells(HUVECs) and Human keratinocyte cells (HaCaT) cells to assess cell proliferation, migration, and angiogenesis. Confocal microscopy was used to track cellular uptake. Activation of the Akt/eNOS pathway and expression of key genes related to inflammation and matrix remodeling were evaluated by Western blotting and qPCR. DDNVs significantly accelerated wound healing and promoted angiogenesis in vivo, as evidenced by enhanced CD31 expression and collagen remodeling. In vitro, DDNVs entered cells efficiently and stimulated HUVEC and HaCaT proliferation and migration. This was accompanied by activation of the Akt/eNOS signaling pathway, increased expression of eNOS and VEGFR-2, upregulation of extracellular matrix(ECM) components (Vimentin, Fibronectin, COL1A1), and suppression of inflammatory markers such as ICAM-1 and IL-1β. DDNVs exhibit strong potential to enhance skin wound healing by promoting angiogenesis, improving tissue repair, and modulating inflammation. These findings support the clinical development of DDNVs as a novel, plant-derived nanotherapeutic for chronic wound treatment and skin regeneration.

**Supplementary Information:**

The online version contains supplementary material available at 10.1186/s40643-025-00915-3.

## Introduction

Wound healing is a regulated, multi-phase process encompassing hemostasis, inflammation, cell proliferation, tissue formation, and remodeling. Under normal physiological conditions, macrophages migrate to the wound site during the hemostasis and inflammation phases to phagocytose bacteria and clear damaged tissue, a process generally completed within 72 h (Wang et al. 2021a, b). Next is the proliferative phase, characterized by the proliferation of cells and connective tissues, leading to the formation of new granulation tissue and blood vessels (Karaman et al. 2018). Angiogenesis represents a critical phase that generally transpires within several hours to four days following tissue injury. During this process, growth factors released by platelets, the extracellular matrix, and macrophages stimulate endothelial cells. This stimulation induces the secretion of proteases by endothelial cells, which allow endothelial cells to migrate and form new capillaries (Karaman et al. 2018; Su et al. 2023). Subsequently, these nascent blood vessels mature, providing a structural scaffold for collagen deposition and tissue remodeling, which supports the coordinated progression of wound healing (Burgess et al. 2021). In pathological conditions, such as chronic ulcers, the healing process is often disrupted by persistent inflammation. This sustained inflammatory state impairs endogenous growth factor expression, limits endothelial cell proliferation, and reduces angiogenesis. As a result, re-epithelialization is delayed, keratinocyte formation is hindered, and fibroblast function is compromised (Singh et al. 2022). Additionally, the absence of a functional vascular network leads to poor oxygen and nutrient delivery, inefficient waste removal, and impaired microcirculation, all of which further delay wound resolution (Chao et al. 2021).

Although growth factor-based therapies, such as bFGF, VEGF, and PDGF, have demonstrated clinical promise, their widespread use remains limited due to concerns regarding drug resistance, oncogenic potential, and cost. Only a few approved formulations are currently available, and they do not meet the large-scale clinical need (He et al. 2024).In contrast, traditional Chinese medicine (TCM) and naturally derived products offer a cost-effective and safer alternative, owing to their multi-component and multi-target pharmacological actions (Berry-Kilgour et al. 2021). These natural compounds demonstrate substantial efficacy in the treatment of conditions such as skin ulcers by modulating endogenous growth factors and signaling pathways. For example, active constituents in traditional Chinese medicine have been shown to enhance endothelial cell proliferation and migration, improve vascular permeability, and facilitate wound healing through the modulation of pathways including PI3K/Akt, Ang-2/Tie2, and p38 MAPK/NF-κB (Fan et al. 2024).

Furthermore, these components inhibit the expression of inflammatory mediators, facilitate the release of anti-inflammatory factors, and modulate nitric oxide signaling, thereby engaging in oxidative stress and inflammatory responses to maintain homeostasis in inflammation (Stark et al. 2018). In addition, they upregulate the expression of TGF-β, α-SMA, and collagen types I, thereby promoting tissue remodeling and facilitating wound healing (Wang et al. 2018). Consequently, traditional Chinese medicine and natural products demonstrate distinct advantages and significant potential in enhancing wound healing.

*Dendrobium officinale*, a highly revered orchid species in traditional Chinese medicine, boasts a medicinal history spanning thousands of years. During the *Tang Dynasty*, it was honored as the most prestigious of the *“Nine Immortal Herbs”* in the *Taoist Canon*, underscoring its historical significance (Wang et al. 2021a, b). In traditional Chinese medicine, the challenges associated with skin wound healing are frequently ascribed to the presence of internal heat toxins and a deficiency of Qi, which typically manifest as inflammation and infection (Kang et al. 2021; Li et al. 2020), *Dendrobium officinale* has been shown to mitigate fluid loss induced by heat-related ailments and to facilitate wound cleansing and tissue regeneration. Furthermore, it enhances angiogenesis, thereby offering substantial support for wound healing (Zuo et al. 2020).

Plant-derived nanovesicles (PDNV) demonstrate biocompatibility and targeting capabilities (Hou et al. 2024), enabling the delivery of proteins, lipids, and bioactive molecules such as miRNA (Subha et al. 2023; Yang et al. 2024). These nanovesicles have the capacity to stimulate angiogenesis by mimicking intercellular communication, which is a critical factor in wound healing and tissue regeneration (Tan et al. 2024a, b), They facilitate the proliferation, migration, and re-epithelialization of skin wound cells (Huang et al. 2023; Cao et al. 2023), Similar to exosomes found in animal systems, these nanovesicles influence key signaling pathways, including VEGF, PKA, and Wnt/β-catenin, thereby promoting angiogenesis (Chen et al. 2024). Additionally, plant-derived nanovesicles extracted from sources such as ginseng (Tan et al. 2024a, b), cucumber (Abraham et al. 2022), tomato (Daniello et al. 2024) and dandelion (Tan et al. 2024a, b) further substantiate their potential in skin repair.

*D. officinale* is particularly rich in polysaccharides and flavonoids, which provide anti-inflammatory, antioxidant, and Yin-nourishing effects. These pharmacological attributes are closely aligned with traditional Chinese medicine concepts of clearing “heat-toxins” and replenishing fluids, which are especially relevant in the treatment of inflammatory and non-healing cutaneous wounds. Such characteristics provide a strong theoretical and phytochemical foundation for the potential therapeutic application of Dendrobium officinale-derived nanovesicles (DDNVs) in wound healing.

Our previous findings have shown that DDNVs significantly downregulate pro-inflammatory cytokine IL-1β expression (Tu et al. 2024), and may influence immune cell infiltration and cellular senescence pathways—both critical factors in chronic wound pathophysiology. These biological actions suggest that DDNVs may possess broader immunoregulatory and regenerative capabilities compared to other plant-derived extracellular vesicles (PDNVs), such as those from ginseng, tomato, or dandelion. However, despite their promising preliminary effects, the precise molecular mechanisms by which DDNVs exert their wound-healing functions remain largely undefined.

**Fig. 1:**
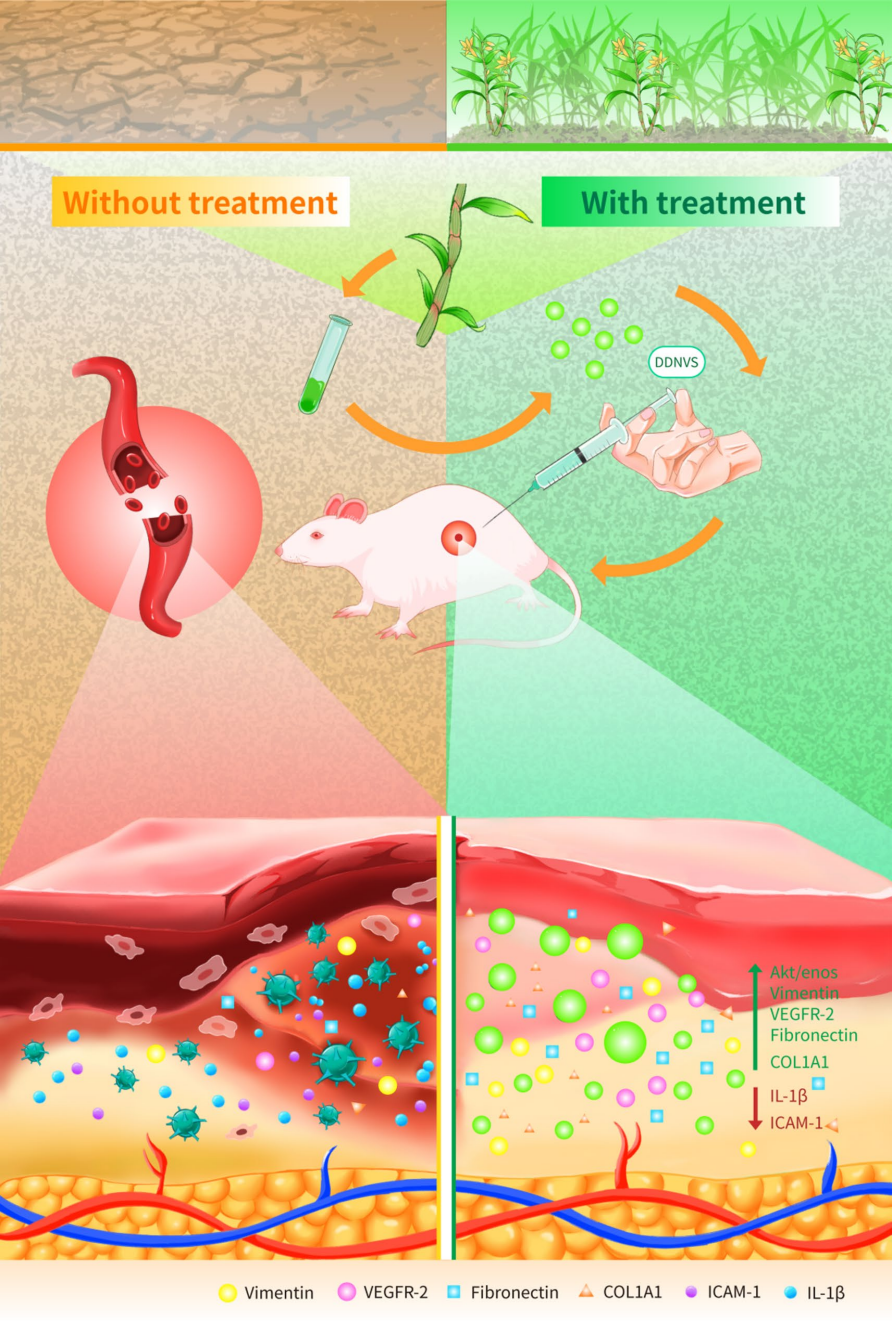
The schematic shows DDNVs’ role in enhancing neovascularization and tissue repair while reducing inflammation in skin wounds

This study aims to explore the therapeutic functions and mechanisms of DDNVs in wound repair. By integrating in vitro and in vivo models, we will assess their effects on angiogenesis, extracellular matrix remodeling, and inflammation resolution (Fig. 1). These findings will lay the groundwork for the clinical translation of DDNVs as a novel therapy for cutaneous wound healing.

## Materials and methods

### Materials

The SteadyPure Rapid RNA Extraction Kit, Evo M-MLV Mixing Kit, and Q-PCR Kit were procured from Acres Bioengineering Co., Ltd, located in Hunan, China. Dewaxing solution, hematoxylin and eosin(HE) dye kit, and Masson dye kit were acquired from Servicebio Technology Co., Ltd, based in Wuhan, China. Antibodies targeting Vimentin, ICAM-1, VEGFR-2/KDR, α-tubulin, Collagen Type I, eNOS, and p-eNOS (Thr495) were obtained from Sanying Biotechnology Co., Ltd, also in Wuhan, China. Additionally, antibodies for Akt and p-Akt (Ser473) were sourced from Cell Signaling Technology, situated in Massachusetts, USA. HUVEC cells and HaCaT cells were procured from iCell Bioscience Inc, located in Shanghai, China.

### Extraction and purification of dendrobium nanovesicles

The fresh Dendrobium stems utilized in this study were procured from Huoshan County, Lu’an City, Anhui Province, China. Botanical identification conducted by Zhao Xinlei verified the specimens as belonging to the Dendrobium species within the Orchidaceae family. The samples were cataloged in the Shanghai Censhan Herbarium (CSH) under the barcode CSH0070204. DDNVs were extracted and purified through a multi-step procedure. Initially, the Dendrobium stems, harvested in spring, underwent a triple rinse with purified water, followed by air-drying. Subsequently, the stems were mechanically processed using a wall-breaker. The processed material was then immersed in purified water for a duration of three minutes.The supernatant was sequentially centrifuged at 300 g for 10 min, 2,000 g for 20 min, and 10,000 g for 30 min. It was then centrifuged at 135,000 g for 70 min and resuspended in 20 mmol/L Tris-HCl, resulting in a green suspension enriched with DDNVs. This suspension underwent gradient centrifugation with sucrose solutions at 15%, 30%, 45%, and 60%, followed by final centrifugation at 150,000 g for 3 h to isolate the 60% fraction. Sucrose was eliminated by resuspending the isolated fraction in 20 mmol/L Tris-HCl, followed by centrifugation at 150,000 g for 70 min. The supernatant was discarded, and the purified DDNVs were obtained by resuspending the pellet in 20 mmol/L Tris-HCl. Subsequently, the DDNVs were sterilized using a 0.22 μm filter, resulting in a sterile suspension. The protein concentration of the suspension was quantified utilizing a BCA assay kit.To ensure reproducibility and maintain biological activity, all DDNVs suspensions used in this study were freshly prepared and stored at 4 °C, with a storage duration not exceeding seven days. This protocol was chosen based on evidence that short-term refrigeration is sufficient to preserve vesicle integrity and protein function in plant-derived extracellular vesicles (PDEVs). For example, Extracellular vesicles(EVs) derived from Dendropanax morbifera retained their morphology and uptake capacity when stored cold, while extended exposure to ambient temperature or repeated freeze–thaw cycles led to significant degradation (Kim et al. 2022).

### Characterisation of Dendrobium ferrugineum nanovesicles

#### Determination of particle size of plant nanovesicles

A pre-diluted (1:100) solution was employed to assess the particle size of plant-derived exosomes. The solution was allowed to equilibrate at room temperature for 2 min to ensure sample stabilization. Following equilibration, the particle size of the exosomes was measured using Nanoparticle Tracking Analysis (NTA) with a ZetaView PMX-120 (Particle Metrix, Germany).

#### Transmission electron microscopy observation of nanovesicle morphology and size

The pre-diluted nanovesicle samples were prepared and deposited onto a copper mesh supported by filter paper. A 10 µL aliquot of the nanovesicle suspension was applied to the copper mesh and allowed to stand for 10 min.A drop of phosphate-buffered saline (PBS) was applied, followed by air-drying at ambient temperature. The samples were subsequently examined using a transmission electron microscope (TEM: FEI, Tecnai G2 12, USA), and images were acquired to document the morphology and size of the nanovesicles.

### Animal experiment section

#### Uptake of skin tissue nanovesicles

Eight-week-old male BALB/c mice were anesthetized with 0.3% sodium pentobarbital (60 mg/kg) and had their dorsal fur shaved. They received subcutaneous injections of 200 µL Tris-HCl (negative control) and 200 µL PKH26-labeled DDNVs (10 mg/mL protein). The following day, skin tissues from the PKH26-DDNV-injected mice were collected, frozen, and sliced into 10 μm cryosections.The distribution and localization of PKH26-labeled DDNVs within skin tissues were evaluated via DAPI staining (10 µg/mL), and fluorescence images were obtained for signal analysis.

#### Measurement of skin wound healing

Male BALB/c mice were anesthetized, and their dorsal hair was removed using an electric razor.The skin of each mouse was disinfected using 75% ethanol, followed by the creation of a circular full-thickness excision wound with a diameter of 6 mm on the dorsal surface. The mice were randomly divided into two groups (*n* = 5 per group): Control group, which received a subcutaneous injection of 200 µL of 20 mmol/L Tris-HCl; DDNVs group, which received a subcutaneous injection of 200 µL of DDNVs with a protein concentration of 10 mg/mL (Pomatto et al. 2023; Saroj et al. 2025). Injections were administered daily, and wound healing was monitored with measurements taken on days 0, 3, 6, and 10.The wound healing rates in both experimental groups were compared to assess the impact of DDNVs on the wound healing process.

The wound healing rate, expressed as a percentage, was determined using the formula: Wound healing rate (%) = [(A₀ - Aₜ) / A₀] × 100, where A₀ denotes the initial wound area and Aₜ represents the wound area on days 3, 6, or 10 post-operation.

#### Immunofluorescence staining of mouse skin tissue

On the tenth day of DDNV treatment, wound tissues were harvested from both the control and DDNV-treated cohorts through full-thickness dermal excision, extending 2 mm beyond the wound margins. The excised tissues were subsequently fixed in 4% paraformaldehyde, followed by dehydration and paraffin embedding after a series of ethanol and xylene treatments. Post-embedding, 10-µm-thick sections were prepared using a microtome. These sections were then subjected to CD31 immunofluorescence staining and DAPI nuclear staining. Following the staining procedure, the tissue sections were sealed, imaged, and examined using a fluorescence microscope. A semi-quantitative analysis of CD31 immunofluorescence was conducted utilizing ImageJ software.

The mean gray value, representing the mean fluorescence intensity, was determined using the formula: Mean Gray Value = Sum of Fluorescence Intensity (Integrated Density) / Area.

#### Histological observations

Tissue samples from the peripheral region of the wound were harvested on the 10th day following injury. These samples underwent fixation in 4% paraformaldehyde, followed by dehydration through a graded ethanol series, and were subsequently embedded in paraffin wax. The paraffin-embedded tissues were sectioned into slices with a thickness of 10 μm, oriented perpendicularly to the wound surface.

The tissue sections were stained using hematoxylin and eosin (HE) and Masson’s trichrome, employing Servicebio kits.After staining, the sections were examined microscopically, and images were captured for analysis.

### Cell culture

This investigation employed primary HUVECs and HaCaT. The cells were rapidly thawed in a 37 °C water bath, sterilized with 75% ethanol, and subsequently transferred to a laminar flow hood for recovery. Upon reaching approximately 80% confluence in the culture flask, the cells were passaged. The cells were then collected into a sterile centrifuge tube, centrifuged, and the supernatant was discarded.Following staining, the sections underwent microscopic examination, and images were acquired for subsequent analysis.

### Cellular nanovesicle uptake

#### Exosome labeling

In accordance with the manufacturer’s protocol, 10 mg of DDNVs were initially combined with Diluent C to remove any unbound exosomes. Subsequently, 2 µL of PKH26 stain was added to 500 µL of Diluent C to formulate the PKH26 working solution. This solution was then mixed with the 10 mg of DDNVs and incubated at ambient temperature for 30 min. The resulting mixture was transferred into sterile ultracentrifuge tubes and subjected to centrifugation at 100,000 g for 1 h. Following centrifugation, the supernatant was discarded, and the labeled exosomes were resuspended in 1 mL of Diluent C.An appropriate volume of complete medium was added to achieve uniform resuspension of the cells, which were then incubated for continued culture. The resuspended exosomes were aliquoted into dark, sterile Eppendorf tubes and stored at -80 °C.

#### Cellular uptake of PKH26-DDNVs

HUVEC cells were seeded in confocal dishes at a density of 1 × 10^5 cells per well, while HaCaT cells were seeded at a density of 1.6 × 10^5 cells per well. Following a 24-hour incubation period, the cells reached approximately 50% confluence. At this juncture, PKH26-labeled DDNVs were introduced at a concentration of 2.5 × 10^8 particles/mL. The cells were then co-cultured with the DDNVs for an additional 24 h. Subsequently, the cell nuclei were stained with DAPI, and the uptake of DDNVs was evaluated using ultra-high-resolution laser confocal microscopy.

#### EdU

HUVECs were seeded at 1 × 10^5 cells per well in 12-well plates. Upon reaching 70% confluence, they were treated with DDNVs for 6 h at concentrations of 5 × 10^7, 2.5 × 10^8, and 1 × 10^9 particles/mL, along with a control group. After treatment, the medium was refreshed with ECM. At 85% confluence, EdU staining was conducted using the Beyotime EdU Cell Proliferation Kit.Cells were incubated in a 2× EdU solution for 3 h, washed with PBS, fixed in 4% paraformaldehyde, and stained for nuclei. Images were taken with a fluorescence microscope and analyzed using ImageJ software.

#### Scratch experiment

HUVECs were cultured in 6-well plates and treated as described in Sect. “EdU”. Once cells reached 90% confluence, a uniform scratch was made in the monolayer using a 200 µL sterile pipette tip.Uniform scratches were made, and cells were washed with PBS and 1% ECM medium. HUVEC migration was imaged at 0 and 24 h.

#### Tube-forming experiment

Matrix gel was thawed at 4 °C overnight, and sterile tips and 96-well plates were pre-cooled. HUVECs, treated as per Sect. “EdU”, were seeded in 12-well plates. At 70% confluence, cells were treated with DDNVs for 6 h, after which the medium was replaced with complete ECM medium.After 24 h, 80 µL of matrix gel was placed into pre-cooled 96-well plates and incubated for 30–60 min. Then, 1 × 10^4 cells were added to each well in triplicate, and the plates were incubated at 37 °C for 6 h. Tube formation was imaged with an inverted microscope and analyzed with ImageJ.

### Real-time quantitative PCR (q-PCR) analysis

#### Total RNA extraction

Total RNA was extracted using the SteadyPure RNA kit.RNA samples underwent purification through lysis, centrifugation, and elution, then were stored at -80 °C. cDNA was synthesized via reverse transcription following kit instructions. SYBR Green Master Mix was used for fluorescence detection, and gene expression was measured with qPCR on the CFX96 RT-PCR system.

#### Real-time quantitative fluorescent detection

Each well contained a 20 µL reaction mixture prepared in a sterile setting. Quantitative fluorescence detection was conducted with SYBR Green Master Mix and analyzed on the CFX96 RT-PCR system. Gene primers are listed in Table 1.

**Table 1:** Primer sequences of related genes

Species	Target	Forward primer (5’->3’)	Reverse primer (5’->3’)
Homo	β-actin	CATGTACGTTGCTATCCAGGC	CTCCTTAATGTCACGCACGAT
Homo	fibronectin	CGGTGGCTGTCAGTCAAAG	AAACCTCGGCTTCCTCCATAA
Homo	vimentin	AGTCCACTGAGTACCGGAGAC	CATTTCACGCATCTGGCGTTC
Homo	eNOS	TGATGGCGAAGCGAGTGAAG	ACTCATCCATACACAGGACCC
Homo	VEGFR-2	GTGATCGGAAATGACACTGGAG	CATGTTGGTCACTAACAGAAGCA
Homo	Icam1	ATGCCCAGACATCTGTGTCC	GGGGTCTCTATGCCCAACAA
Homo	IL-1β	ATGGCATGAACTGGGTCCG	CTCTGGAGATGGTGAATCGGC

#### Western blot

Total protein was extracted from cells treated with different DDNV concentrations, washed with PBS, lysed, and centrifuged. Protein concentration was determined using the BCA assay. Samples were mixed with loading buffer, heated for denaturation, and analyzed via SDS-PAGE.Proteins were transferred to PVDF membranes, washed with TBST, and incubated overnight with primary antibodies. After further washes, secondary antibodies were applied, and protein bands were detected via chemiluminescence imaging. Band intensity was quantified using ImageJ.

### Statistical analysis methods

Experimental data were analyzed with at least three independent replicates using GraphPad Prism 9.0. One-way ANOVA and Dunnett’s test were applied to normally distributed data, while the Kruskal-Wallis H test and Bonferroni-corrected pairwise comparisons were used for non-normally distributed data. A p-value below 0.05 indicated statistical significance.

## Results

### Isolation and characterization of DDNVs

**Fig. 2:**
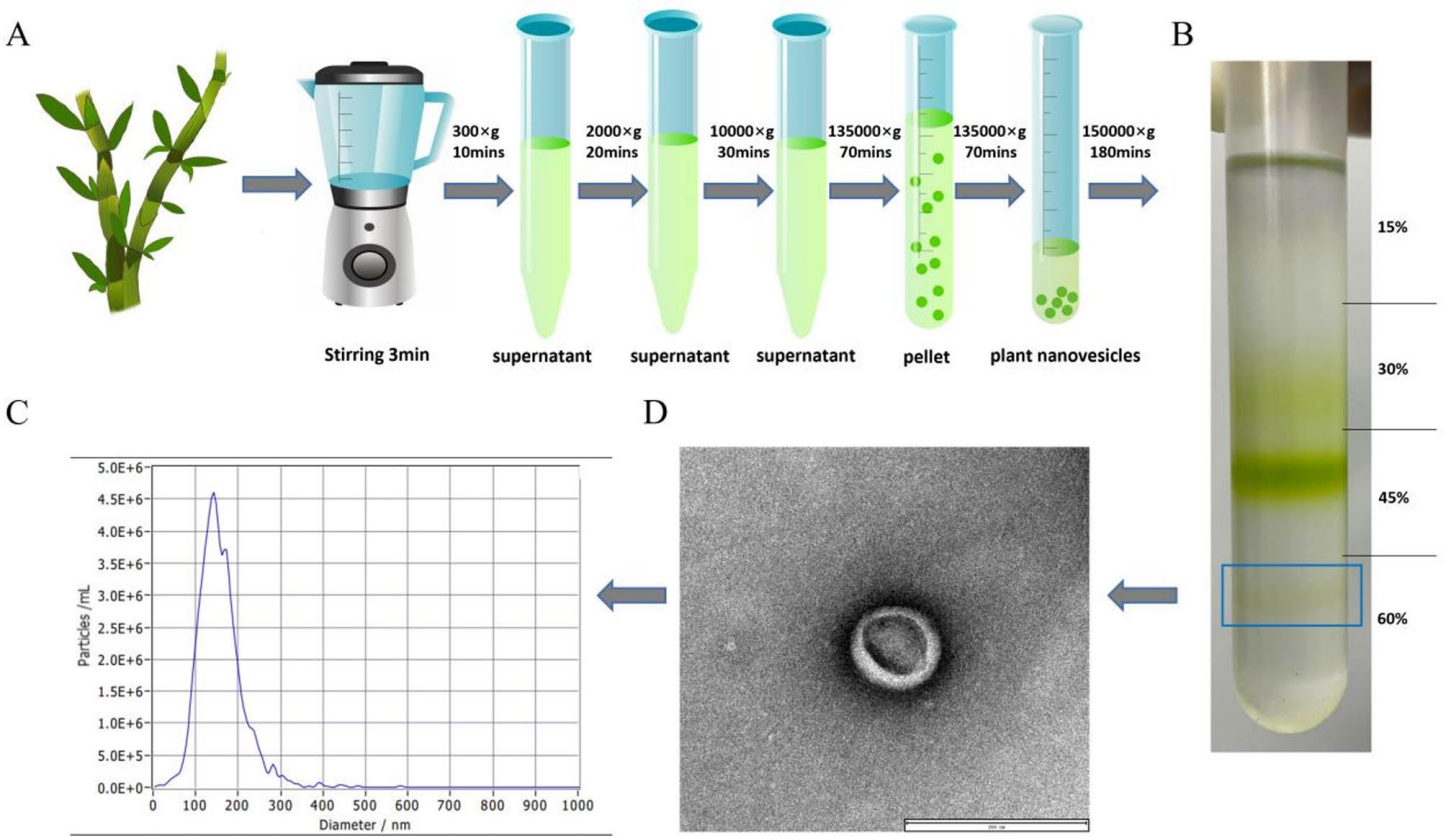
Extraction, Characterization, and Analysis of DDNVs. (**A**) Extraction of Dendrobium-derived DDNVs using different centrifugal gradients. (**B**) DDNV purification was achieved through differential centrifugation followed by sucrose gradient ultracentrifugation. During ultracentrifugation, the sucrose gradient was divided into four layers with concentrations of 15%, 30%, 45%, and 60%, from top to bottom. After ultracentrifugation, DDNVs were primarily localized in the 60% sucrose gradient. (**C**) Transmission electron microscopy images of Dendrobium officinale-derived nanovesicles collected from the 60% sucrose gradient layer. (**D**) The size and concentration of DDNVs were analyzed using nanoparticle tracking analysis (NTA) (Scale bar = 200 nm)

After 3 h of centrifugation, nanovesicles were mainly found in the 30%, 45%, and 60% sucrose density layers (see Fig. 2A and B). Figure 2B shows the purified DDNVs. Exosomal protein concentration was determined using the BCA protein assay, measuring absorbance at 562 nm against a standard curve. TEM images showed the nanovesicles had a uniform shape with a clear lipid bilayer.They mainly displayed a saucer-like or hemispherical shape, consistent with typical plant-derived nanovesicles. Nanoparticle tracking analysis (NTA) showed that the purified DDNVs had a mean size of about 141.0 nm, demonstrating high uniformity, as depicted in Fig. 2C and D. The concentration of DDNVs was determined to be 9.4 × 10^9 particles/mL, with NTA data showing a uniform distribution.

### Uptake of nanovesicles in skin tissue

**Fig. 3:**
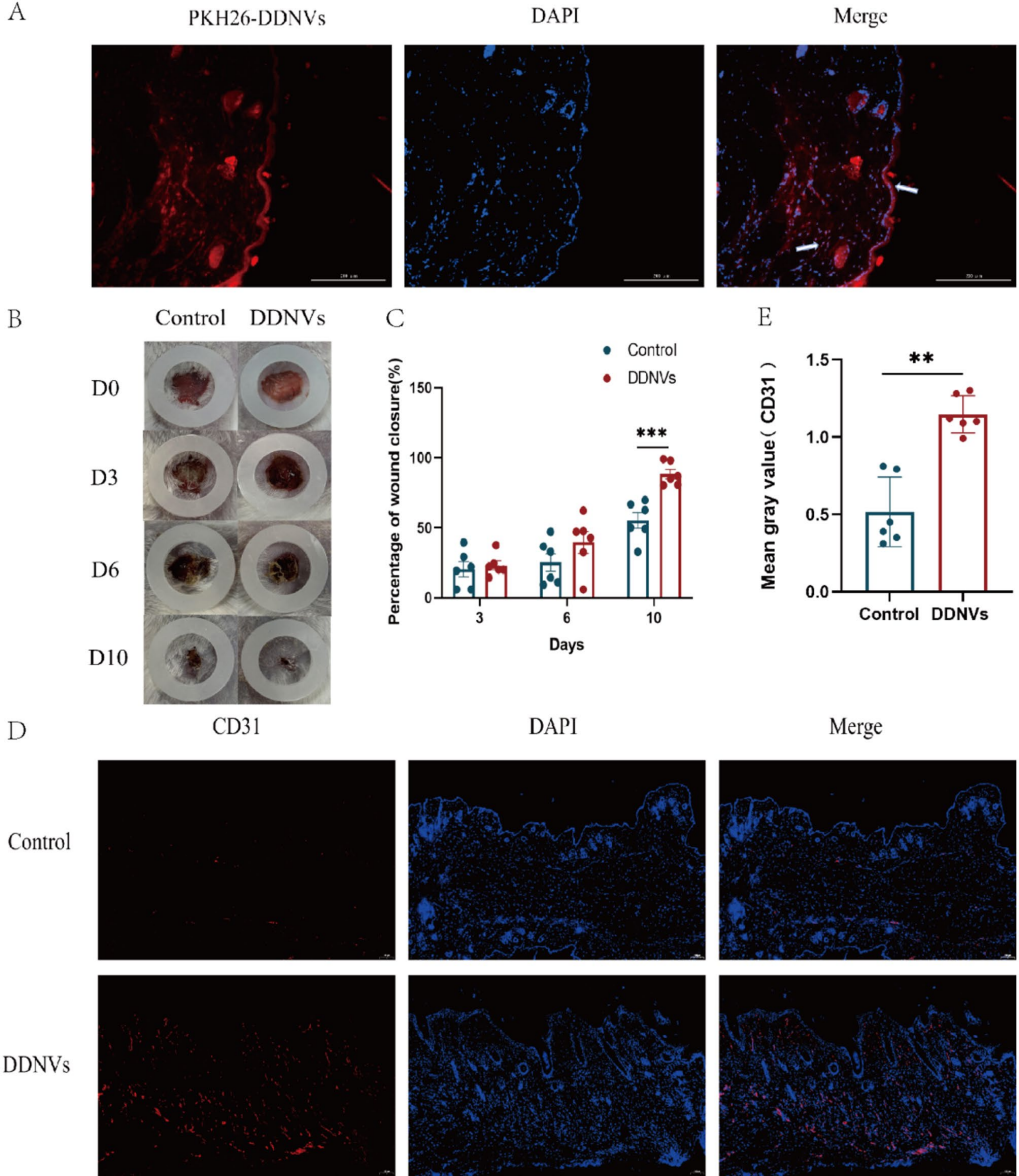
Effects of Local Application of DDNVs on Skin Wound Healing in Mouse Models. (**A**) PKH26-labeled DDNVs were injected into the dorsal skin of mice, and tissue samples were collected 24 h post-injection. PKH26-labeled DDNVs appeared red, while the nuclei were stained blue(Scale bar = 200 μm). (**B**) Full-thickness skin excision wounds were created on the dorsal side of mice, followed by daily treatment with 200 µL of DDNVs (10 mg/mL) until complete wound closure was achieved. (**C**) Percentage of wound healing in mice over the observation period. (**D**) Immunofluorescence staining of CD31 in mouse skin tissue, with CD31 displayed in red and nuclei stained blue(Scale bar = 100 μm. (**E**) Quantitative analysis of the average fluorescence intensity of CD31 staining was performed. Results are based on data from three or more independent experiments. Statistical significance is indicated as *p < 0.05, **p < 0.01 and ***p < 0.001 compared to the control group

To assess in vivo skin uptake, DDNVs were labeled with PKH26 dye and injected subcutaneously at 10 mg/mL into mice dorsal skin. Skin samples were collected 24 h later and frozen for analysis.Fluorescence microscopy demonstrated that DDNVs were effectively internalized by dermal cells, as evidenced by a distinct signal from PKH26-labeled DDNVs within these cells (see Fig. 3A).

### DDNVs enhance skin wound healing in vivo: facilitating tissue repair via CD31 expression and collagen synthesis

**Fig. 4:**
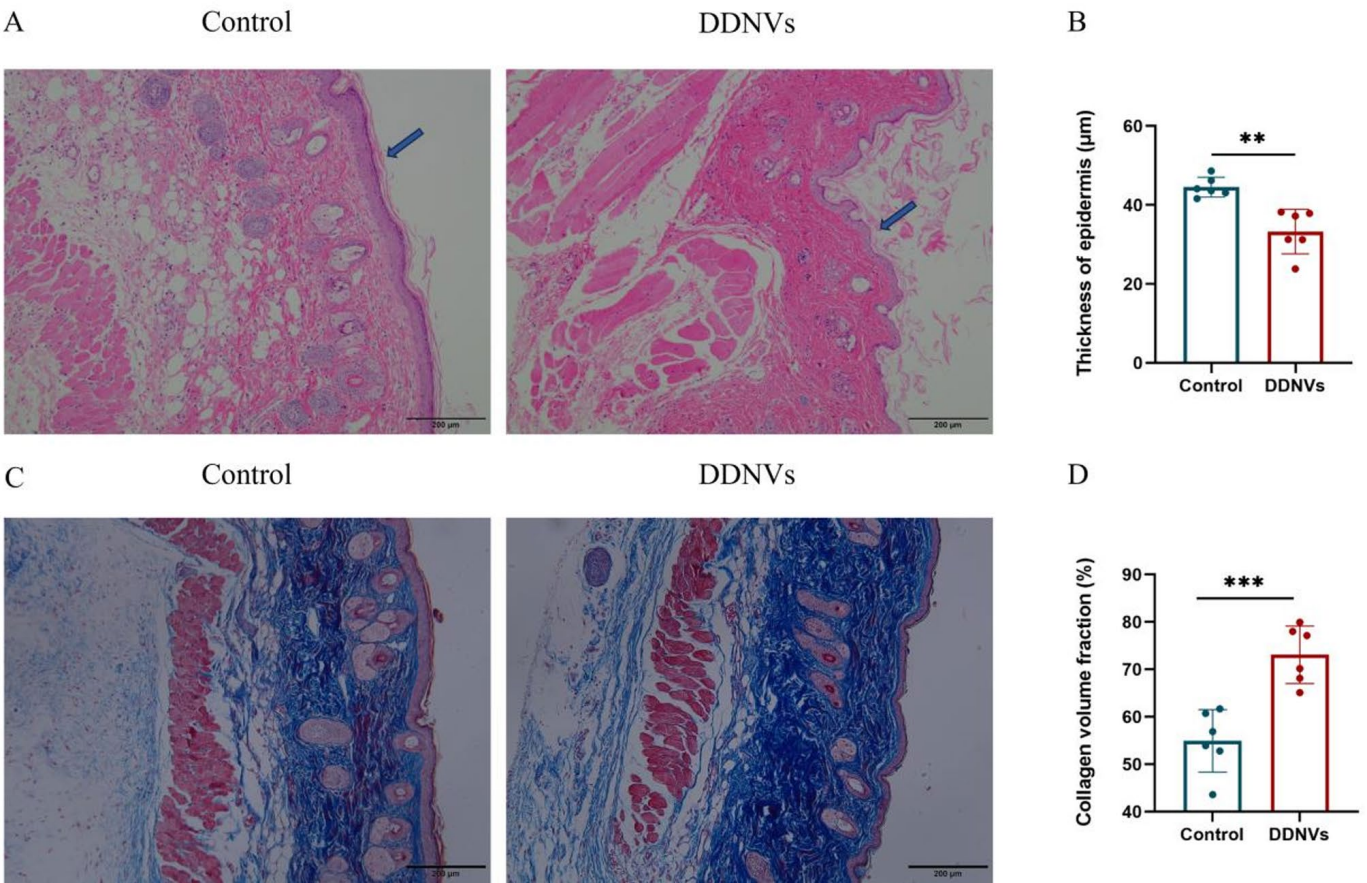
Morphological Analysis of Skin Tissue Following Local Application of DDNVs in Mouse Models. (**A**) HE staining of skin tissue sections to assess tissue morphology, including epidermal thickness following DDNV treatment(Scale bar = 200 μm). (**B**) A statistical graph illustrating epidermal thickness derived from HE-stained skin tissue sections. (**C**) Masson’s staining of skin tissue sections to analyze collagen expression in tissues treated with DDNVs(Scale bar = 200 μm. (**D**) A statistical graph depicting collagen volume analysis derived from Masson-stained skin tissue sections. Results represent data from three or more independent experiments, with statistical significance indicated as **p < 0.01 and ***p < 0.001 compared to the control group

In a skin wound model, subcutaneous injection of DDNVs significantly accelerated wound healing and improved tissue repair over 10 days compared to the control group (see Fig. 3B and C). At key intervals (days 0, 3, 6, and 10), the DDNVs-treated group showed faster wound closure. By day 10, their wounds were almost fully healed, outperforming the control group. Neovascularization, crucial for wound healing, was evaluated on day 10 using CD31 immunofluorescence staining, which marks new blood vessel formation. Immunofluorescence analysis showed that DDNVs-treated tissues had stronger CD31 signals than controls, suggesting increased angiogenesis (see Fig. 3D and E). This highlights DDNVs’ potential to enhance neovascularization in wound healing. On day 10, histological analysis with HE and Masson’s trichrome staining revealed that DDNVs-treated wounds had thinner epidermal layers and more collagen deposition than controls (see Fig. 4). These findings suggest that DDNVs accelerate wound healing by promoting collagen deposition and tissue repair.

### Uptake of nanovesicles in cellular

**Fig. 5:**
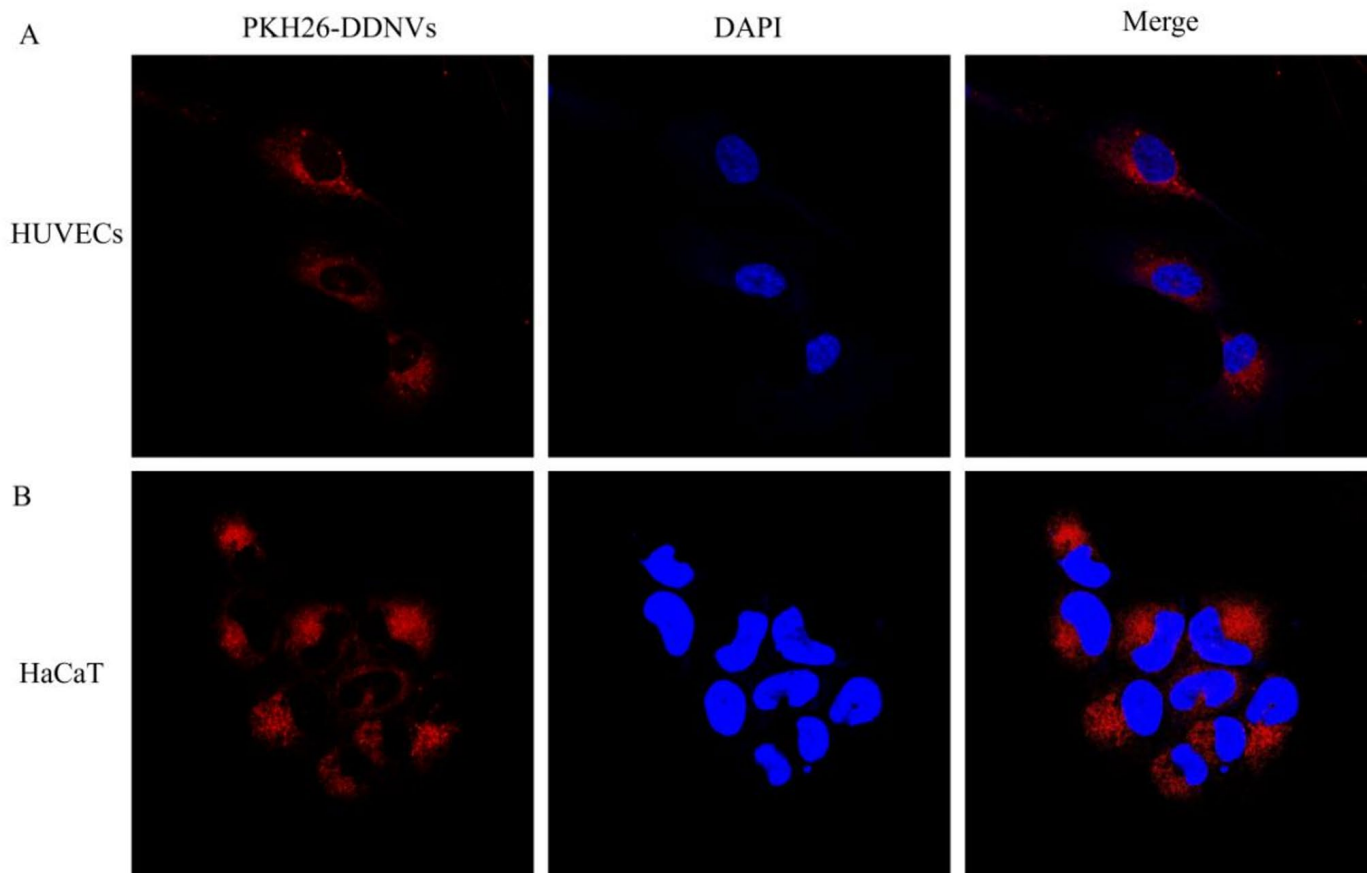
Effects of Cellular Uptake of DDNVs. (**A**) Uptake of PKH26-labeled DDNVs by HUVECs observed at 680x magnification using super-resolution laser confocal microscopy. Red fluorescence indicates the presence of DDNVs within the cells. (**B**) Uptake of PKH26-labeled DDNVs by HaCaT cells observed at 680x magnification using super-resolution laser confocal microscopy. Red fluorescence displays the distribution of DDNVs within the cells

To study the uptake of DDNVs by HUVECs and HaCaT cells, we labeled them with PKH26 dye and co-incubated them with the cells. Super-resolution confocal microscopy showed red fluorescence from the PKH26-labeled DDNVs within both HUVECs and HaCaT cells, indicating their presence on the cell membrane and throughout the cells.This observation shows that DDNVs were effectively internalized by both cell types via endocytosis (see Fig. 5).

### DDNVs promote proliferation of HUVECs and HaCaT cells

**Fig. 6:**
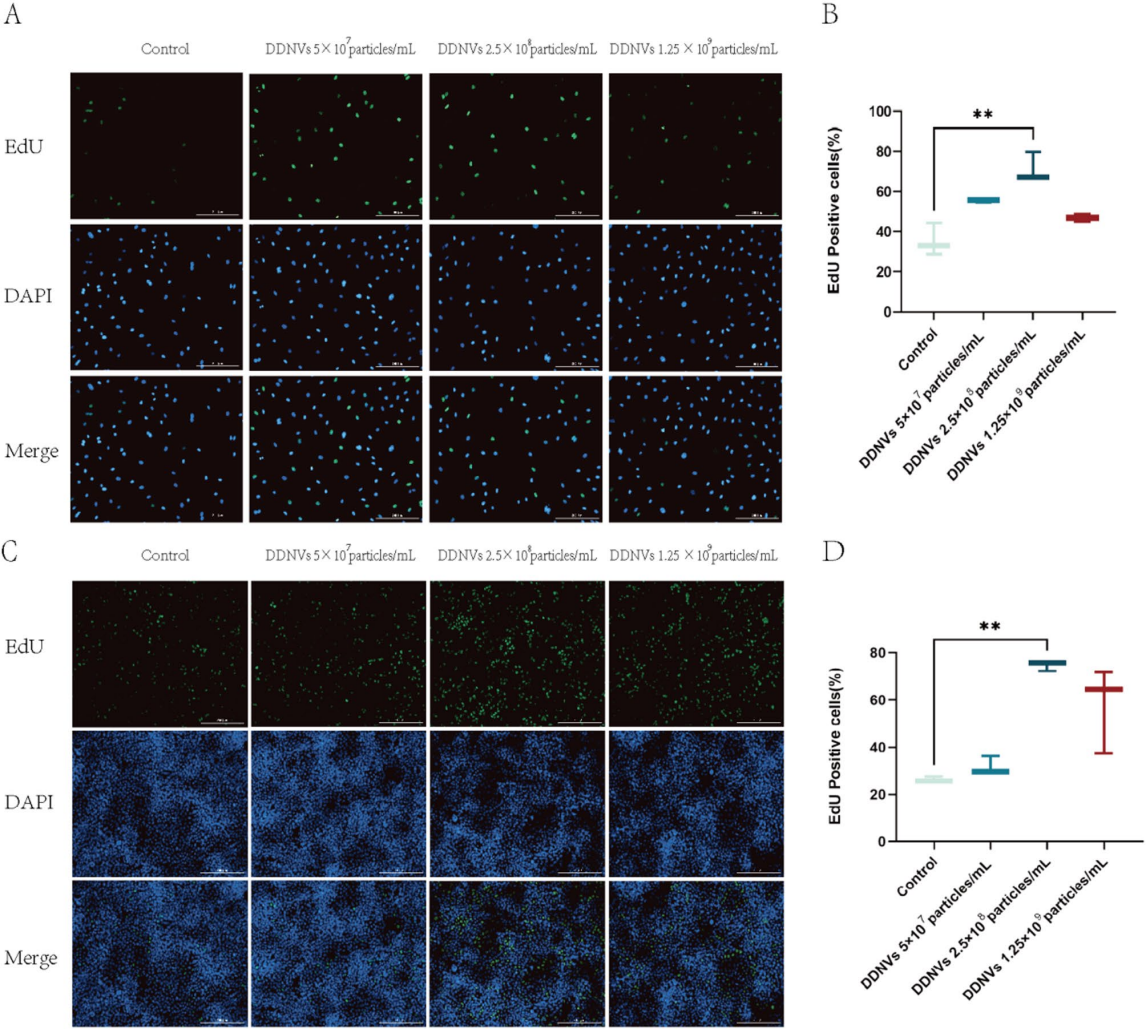
Proliferative Effects of DDNVs on HUVECs and HaCaT Cells. (**A**) The effect of various concentrations of DDNVs on the proliferation of HUVECs. Green fluorescence indicates EdU incorporation, while blue fluorescence highlights the cell nuclei(Scale bar = 200 μm). (**B**) Statistical analysis of the proliferation rates of HUVECs treated with different concentrations of DDNVs. (**C**) The effect of various concentrations of DDNVs on the proliferation of HaCaT cells(Scale bar = 200 μm. (**D**) Statistical analysis of the proliferation rates of HaCaT cells treated with different concentrations of DDNVs. These results represent data from three or more independent experiments with similar outcomes (*n* = 3), indicating statistical significance compared to the control group (**p < 0.01)

We used an EdU assay on HUVECs and HaCaT cells to assess the impact of DDNVs on cell proliferation. Quantitative analysis of EdU-positive cells at various DDNV concentrations demonstrated a significant increase in proliferation in treated groups compared to controls, as revealed by fluorescence microscopy. Figure 6A-D show that DDNVs at 2.5 × 10^8 particles/mL significantly boost proliferation in both HUVECs and HaCaT cells (*p* < 0.01).

### DDNVs enhance cell migration and tube formation

**Fig. 7:**
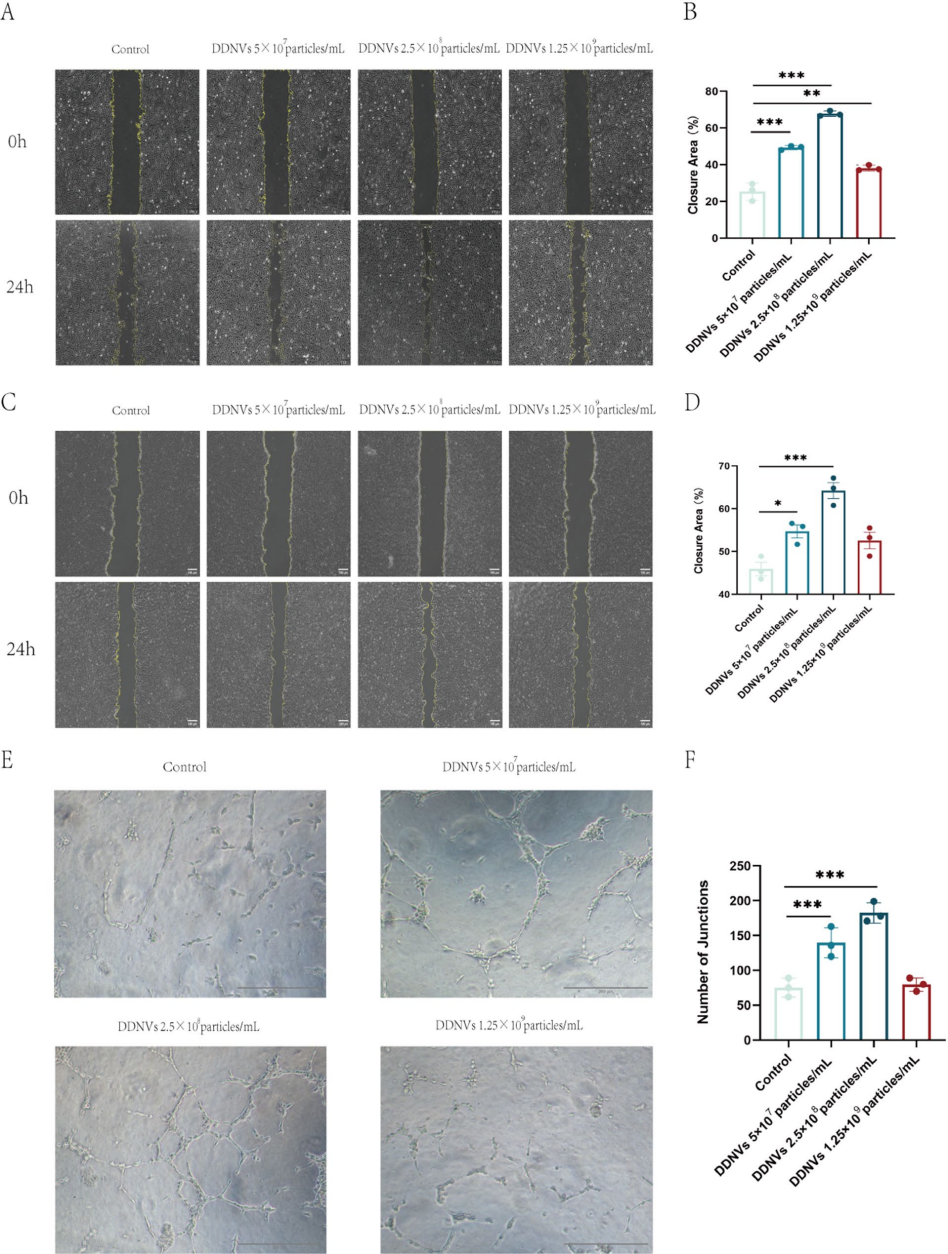
Effects of DDNVs on the Migration Capacity and Angiogenic Activity (**A**) The effect of various concentrations of DDNVs on the migratory capacity of HUVECs after treatment(Scale bar = 100 μm). (**B**) Statistical analysis of the migratory capacity of HUVECs treated with various concentrations of DDNVs. (**C**) The effect of various concentrations of DDNVs on the migratory capacity of HaCaT cells(Scale bar = 100 μm. (**D**) Statistical analysis of the migratory capacity of HaCaT cells after DDNVs treatment. (**E**) The effect of various concentrations of DDNVs on the angiogenic capacity of HUVECs(Scale bar = 200 μm. (**F**) Statistical analysis of the angiogenic capacity of HUVECs treated with various concentrations of DDNVs. These results represent data from three or more independent experiments with consistent outcomes (compared to the control group: *p < 0.05, **p < 0.01, ***p < 0.001)

A wound-healing assay showed that DDNVs enhanced the migration of HUVECs and HaCaT cells. Initially, there were no differences in scratch areas, but after 24 h, DDNV-treated cells exhibited significantly greater closure than controls, as depicted in Figs. 7A–D. To further explore the angiogenic potential of DDNVs, a tube formation assay was performed utilizing HUVECs. The findings demonstrated a substantial increase in tube-like structures in HUVECs treated with DDNVs (see Fig. 7E). In comparison to the untreated control group, the DDNV-treated HUVECs showed a pronounced increase in the number of branch points, indicative of enhanced angiogenesis (see Fig. 7F).

### DDNVs activate the AKT/eNOS signaling pathway and modulate angiogenic gene expression

**Fig. 8:**
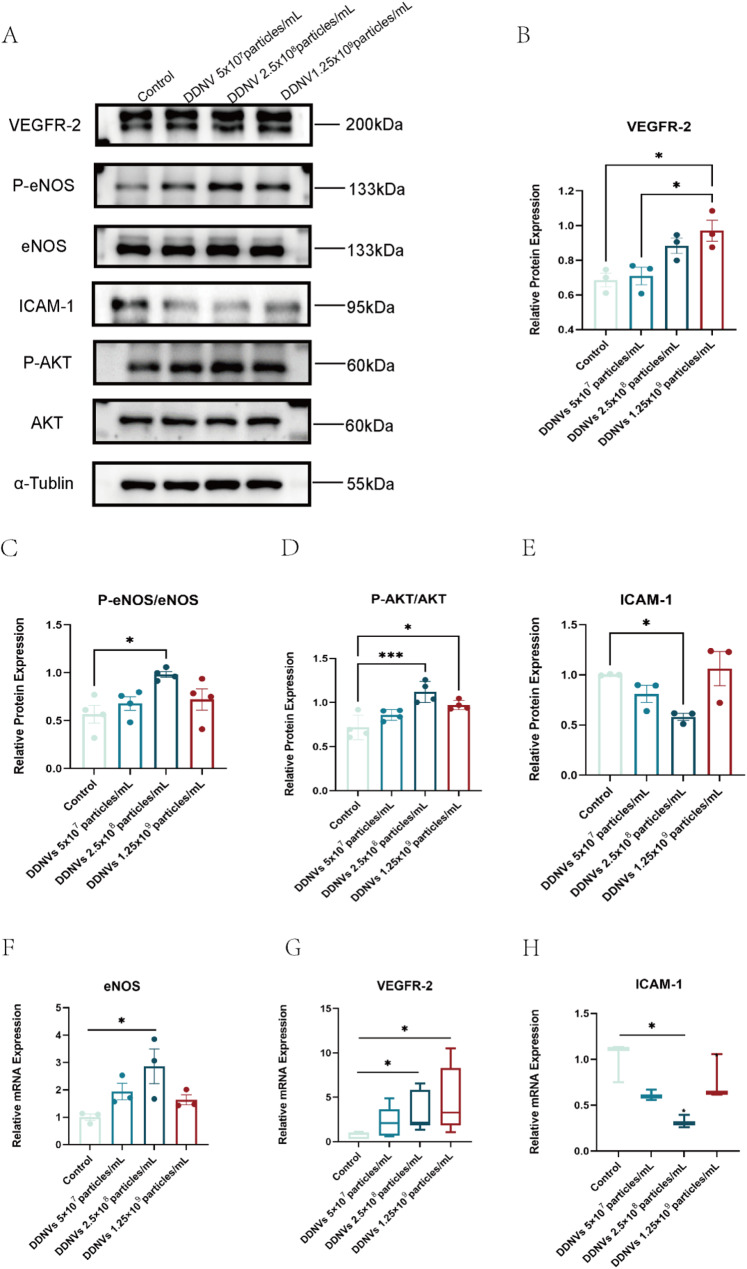
Effects of DDNVs on the Activation of the AKT/eNOS Signaling Pathway and Expression of Angiogenesis-Related Genes in HUVECs. (**A**) Western blot analysis of target proteins involved in the AKT/eNOS signaling pathway. (**B**-**E**) Statistical analysis of the protein levels of AKT, phosphorylated AKT (p-AKT), ICAM-1, eNOS, and phosphorylated eNOS (p-eNOS). (**F**-**H**) Expression levels of eNOS, vascular endothelial growth factor receptor 2 (VEGFR-2, *n* = 7), and intercellular adhesion molecule 1 (ICAM-1, *n* = 3) mRNA in HUVECs following treatment with varying concentrations of DDNVs. These results represent findings from three or more independent experiments and demonstrate consistent outcomes (compared to the control group: *p < 0.05, ***p < 0.001)

This study examined how DDNVs affect key proteins in the AKT/eNOS pathway and the expression of VEGFR-2 and ICAM-1 in HUVECs. DDNVs increased protein phosphorylation in the AKT/eNOS pathway at all concentrations, with the strongest effect at the intermediate level. VEGFR-2 expression rose at low and high concentrations, while ICAM-1 expression decreased significantly at the intermediate concentration (see Figs. 8A).

Statistical analysis showed that DDNVs significantly increase VEGFR-2 expression, activate the AKT/eNOS pathway, and reduce ICAM-1 expression (*p < 0.05, ***p < 0.001) (see Fig. 8B and E). qRT-PCR results in Figs. 8F–H demonstrate that DDNVs (2.5 × 10^8 particles/mL) significantly upregulate eNOS and VEGFR-2 mRNA levels and downregulate ICAM-1 mRNA expression (*p < 0.05) in HUVECs.The study shows that DDNVs boost angiogenesis-related gene expression and reduce inflammation-related gene expression in HUVECs.

### DDNVs upregulate Pro-Wound healing factor expression

**Fig. 9:**
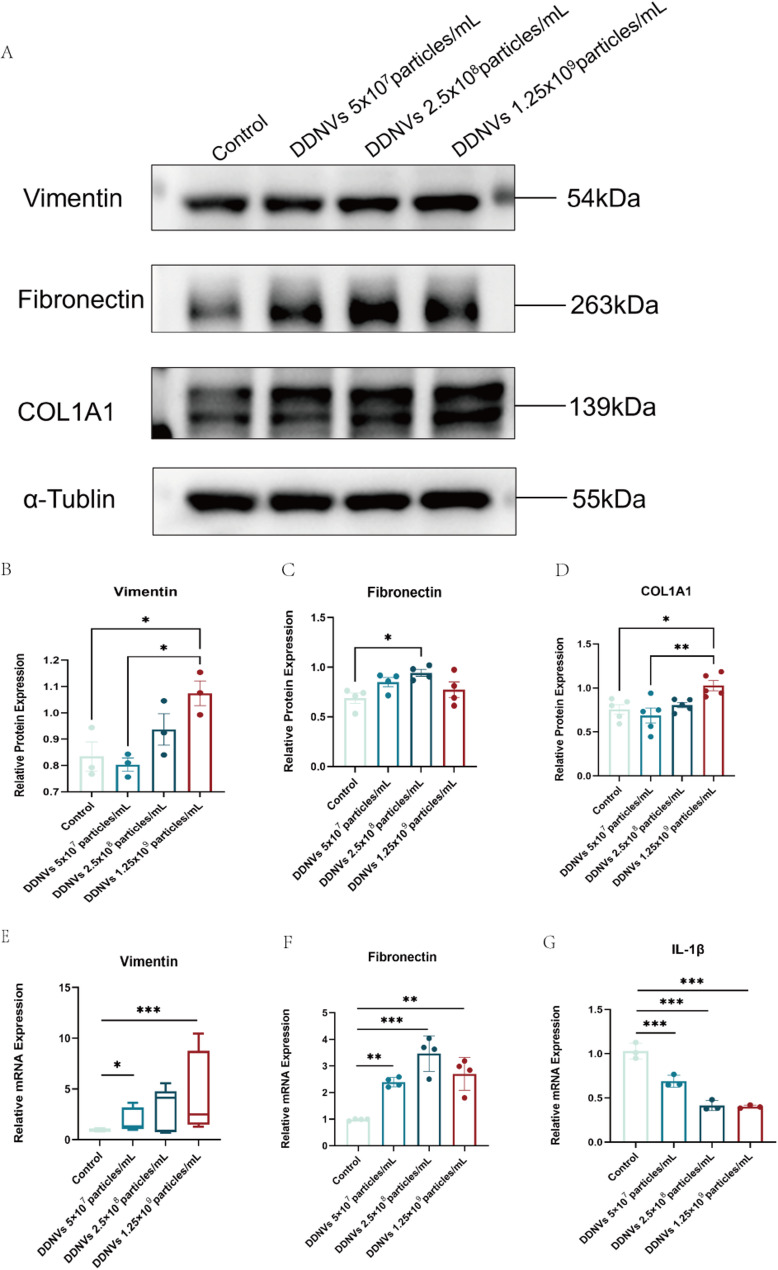
Effects of Dendrobium officinale-Derived Nanovesicles on Wound Healing. (**A**) Western blot analysis demonstrating the expression of key proteins involved in wound healing. (**B**-**D**) Statistical analysis of the protein levels of target proteins associated with wound healing. (**E**-**G**) Expression levels of vimentin (*n* = 8), fibronectin, and interleukin-1 beta (IL-1β) mRNA in HaCaT cells following treatment with varying concentrations of DDNVs. These results represent findings from three or more independent experiments and demonstrate consistent outcomes (compared to the control group: *p < 0.05, **p < 0.01, ***p < 0.001)

In HaCaT cells, DDNVs significantly increased vimentin and COL1A1 protein levels, especially at the highest concentration (10^9 particles/mL), as confirmed by Western blot analysis (*p < 0.05). Treatment with DDNVs at 2.5 × 10^8 particles/mL significantly boosted fibronectin expression (*p < 0.05), indicating that DDNVs enhance key wound healing proteins in HaCaT cells, aiding tissue repair (see Fig. 9A and D). To investigate further, qRT-PCR was used to measure mRNA levels of vimentin, fibronectin, and IL-1β in DDNV-treated HaCaT cells. Figures 9E–G show that high DDNV concentrations (10^9 particles/mL) significantly increase vimentin mRNA (*p < 0.05), while fibronectin mRNA rises at all concentrations. IL-1β mRNA is significantly reduced at low and intermediate levels, with similar effects at the highest concentration. These results indicate that DDNVs influence vimentin, fibronectin, and IL-1β mRNA expression in HaCaT cells, highlighting their role in wound healing regulation.

## Discussion

Extracellular vesicles (EVs) are nanoscale, membrane-bound structures released by various cell types, playing a key role in intercellular and interspecies communication by transferring bioactive molecules such as proteins, lipids, and RNAs (Bahri et al. 2024; Torre-Escudero and Robinson 2017). Plant-derived extracellular vesicles (PDEVs) have recently garnered significant interest for their biomedical potential. Although vesicle-like structures were noted in plants decades ago, systematic efforts to characterize PDEVs as functional nanocarriers have only emerged in recent years (Lo et al. 2024; Nemati et al. 2022). Recent studies indicate that MPEVs possess enhanced biological activity compared to general plant EVs, highlighting their promise as therapeutic agents and drug delivery platforms. The 2023 “Consensus Statement” has further promoted standardization in the research and clinical translation of herbal vesicles (Zhao et al. 2023). Our preliminary studies demonstrate that Dendrobium-derived nanovesicles (DDNVs) significantly promote skin wound healing in murine models, although the precise mechanisms remain to be fully elucidated (Tu et al. 2024).

Endothelial cells and keratinocytes contribute to granulation tissue formation (Tu et al. 2024). Growth factors released by platelets and endothelial cells stimulate keratinocytes, initiating capillary network formation, which facilitates collagen deposition and tissue remodeling essential for wound healing (Chen et al. 2024; Peña and Martin 2024). In this study, we evaluated the therapeutic effects of DDNVs in a murine wound model, and found that they significantly accelerated healing by promoting cellular proliferation and migration at wound edges. A marked increase in neovascularization was observed in the DDNV-treated group, which enhanced local oxygen and nutrient delivery while supporting metabolic waste clearance.

During the healing process, these newly formed vessels help restore circulation, thereby facilitating tissue repair through the proliferation, migration, and differentiation of endothelial cells. Supporting this, our experiments in HUVECs and HaCaT cells showed that DDNVs enhance cell proliferation and migration, suggesting a key role in angiogenesis.

The Akt/eNOS signaling pathway is central to angiogenic regulation (Peña and Martin 2024); specifically, Akt-mediated phosphorylation of eNOS leads to Nitric oxide (NO) production (Yu et al. 2020) NO facilitates vasodilation, promotes endothelial cell growth and motility, and protects against apoptosis—functions essential for neovascular formation (Xu et al. 2023). Our data confirmed that DDNVs significantly enhanced AKT phosphorylation in HUVECs, a pivotal event in activating this pathway. Subsequent upregulation of eNOS activity likely contributed to increased NO synthesis, promoting endothelial proliferation, migration, and vessel formation.Furthermore, quantitative PCR analysis revealed that DDNVs upregulate transcription of both eNOS and VEGFR-2, reinforcing their role in angiogenesis.Collectively, these findings suggest that DDNVs promote angiogenesis by activating the Akt/eNOS signaling axis, enhancing NO production and downstream gene expression relevant to vascular regeneration.

The extracellular matrix (ECM) plays an essential role in wound healing by maintaining tissue integrity, supporting cellular architecture, and regulating cell behavior. In this study, we utilized HE and Masson staining to examine skin tissue morphology and collagen deposition following DDNV treatment. The results demonstrated that DDNVs markedly enhanced overall tissue structure and increased collagen accumulation, suggesting that they may accelerate ECM remodeling by promoting collagen synthesis.

Furthermore, DDNVs significantly upregulated both protein and mRNA expression of key ECM components—Vimentin, Fibronectin, and COL1A1—in HaCaT cells. Vimentin contributes to ECM organization, while Fibronectin and COL1A1 are pivotal for cell adhesion, migration, and proliferation. By elevating these factors, DDNVs likely facilitate ECM remodeling and tissue regeneration, underscoring their therapeutic relevance in wound repair.Inflammation is a critical initial phase in wound healing, acting as the body’s natural response to injury and infection. However, chronic or uncontrolled inflammation can disrupt this process, delaying tissue regeneration. Our findings revealed that DDNVs significantly reduced the expression of inflammatory mediators, particularly IL-1β and ICAM-1, in both HUVECs and HaCaT cells.These anti-inflammatory effects suggest that DDNVs can help restore immune balance at the wound site, thereby creating a more favorable environment for efficient healing.

Current biomedical research is increasingly focused on developing therapies that simultaneously target inflammation, angiogenesis, and tissue remodeling to improve skin wound healing outcomes.Recent advancements suggest that an ideal wound healing agent should possess multifunctional bioactivities across all healing phases. In this context, our study systematically evaluated the multifunctional effects of DDNVs, particularly their roles in promoting angiogenesis, facilitating tissue regeneration, and modulating inflammation.

The findings demonstrated that DDNVs significantly promoted angiogenesis by enhancing endothelial cell function and inducing neovascularization, thereby improving oxygenation, nutrient supply, and metabolic waste removal at the wound site. Additionally, DDNVs enhanced the quality of tissue repair by upregulating extracellular matrix proteins such as Vimentin, Fibronectin, and COL1A1, which are essential for cell adhesion, migration, and proliferation.

Moreover, DDNVs markedly reduced the expression of inflammatory mediators, fostering a more stable, regenerative microenvironment. Collectively, these results highlight that the multifunctional properties of DDNVs provide a strong scientific basis for their potential clinical use. DDNVs appear to accelerate cutaneous wound healing through simultaneous activation of angiogenesis, ECM remodeling, and inflammatory resolution, suggesting promise for the treatment of chronic wounds, post-surgical recovery, and burn repair.

While our study confirms the therapeutic promise of DDNVs, we acknowledge several translational limitations inherent to preclinical models. Specifically, mouse skin differs markedly from human skin in immune architecture, epidermal thickness, and the presence of the panniculus carnosus, which may influence vesicle uptake and biological response. Furthermore, species-specific variations in immune surveillance may affect the compatibility and immunogenicity of plant-derived nanovesicles (PDNVs) in human systems.

Encouragingly, several recent studies have shown that PDNVs—including those derived from Dendrobium officinale—exhibit low immunogenicity and high biocompatibility in mammalian models (Liu et al. 2021), due in part to their natural botanical composition and lack of major histocompatibility complex (MHC) molecules or immunogenic membrane proteins (Li et al. 2023; Zuzarte et al. 2022). For example, ginseng- and grape-derived nanovesicles have been successfully administered intravenously or orally in rodent models without eliciting adverse immune reactions (Wang et al. 2024; Song et al. 2025).

Nevertheless, further validation in human-based systems is essential. Future studies should integrate human skin organoids, full-thickness 3D skin models, or peripheral blood mononuclear cell (PBMC) assays to more accurately evaluate the immunological behavior of DDNVs. These approaches are critical for ensuring safety, optimizing delivery, and elucidating cross-species mechanisms. Ultimately, such efforts will enhance the translational feasibility of PDNV-based therapies for chronic wounds, burns, and surgical recovery.

## Conclusions

This study illustrates that DDNVs markedly facilitate skin wound healing by augmenting angiogenesis, mitigating inflammation, and promoting tissue repair. These findings lay a robust groundwork for the development of DDNVs as a natural therapeutic approach for chronic wounds and offer valuable insights for future clinical and therapeutic applications.

## Electronic supplementary material

Below is the link to the electronic supplementary material.

Supplementary Material 1

## Data Availability

Data is provided within the manuscript or supplementary information files.

## Abbreviations

DDNVs: Dendrobium officinale-derived nanovesicles
PDNV: Plant-derived nanovesicles
HUVECs: Human umbilical vein endothelial cells
HaCaT: Human keratinocyte cells
NO: Nitric oxide
HE: Hematoxylin and eosin
NTA: Nanoparticle Tracking Analysis
PBS: Phosphate-buffered saline
EVs: Extracellular vesicles
ECM: Extracellular matrix
