# Supplementary figures and images for: Dendrobium officinale-derived nanovesicles: a natural therapy for comprehensive regulation of angiogenesis, inflammation, and tissue repair to enhance skin wound healing
Source: Bioresour Bioprocess. 2025 Jul 12;12(1):74. doi: 10.1186/s40643-025-00915-3 (PMC12255648; doi:10.1186/s40643-025-00915-3)

Figure 8-1

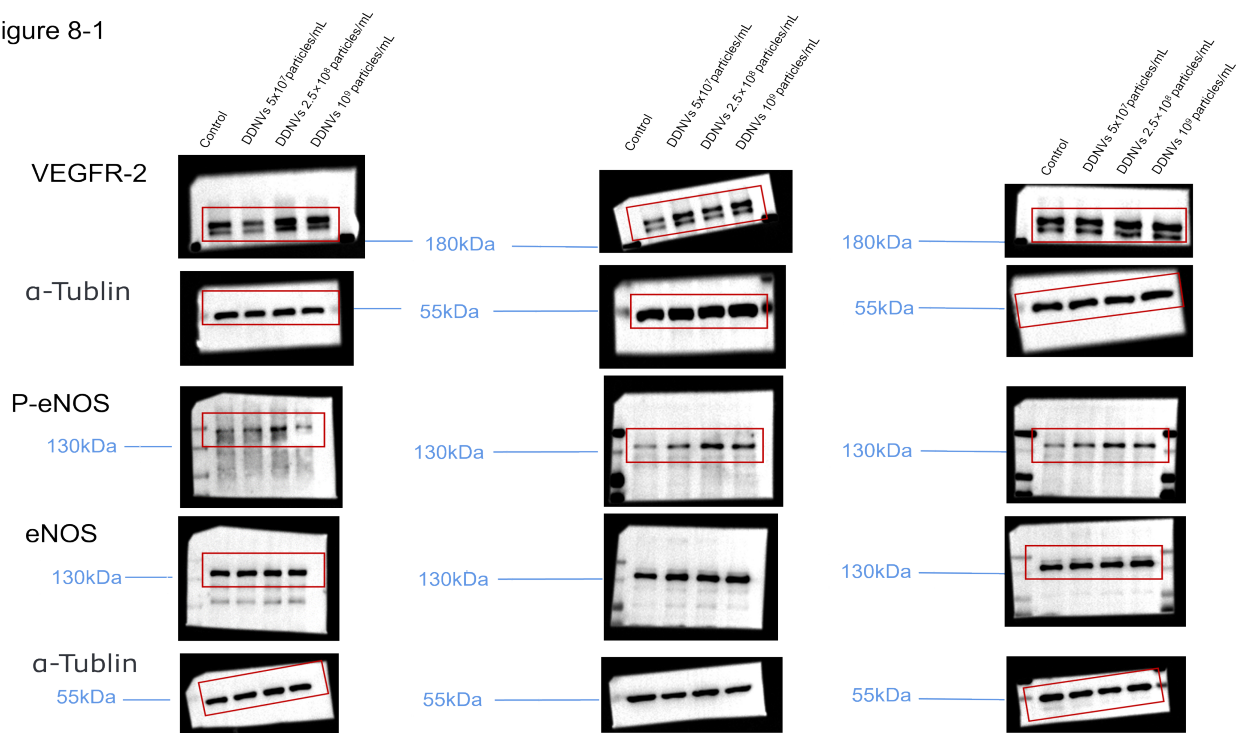

Figure 8-2

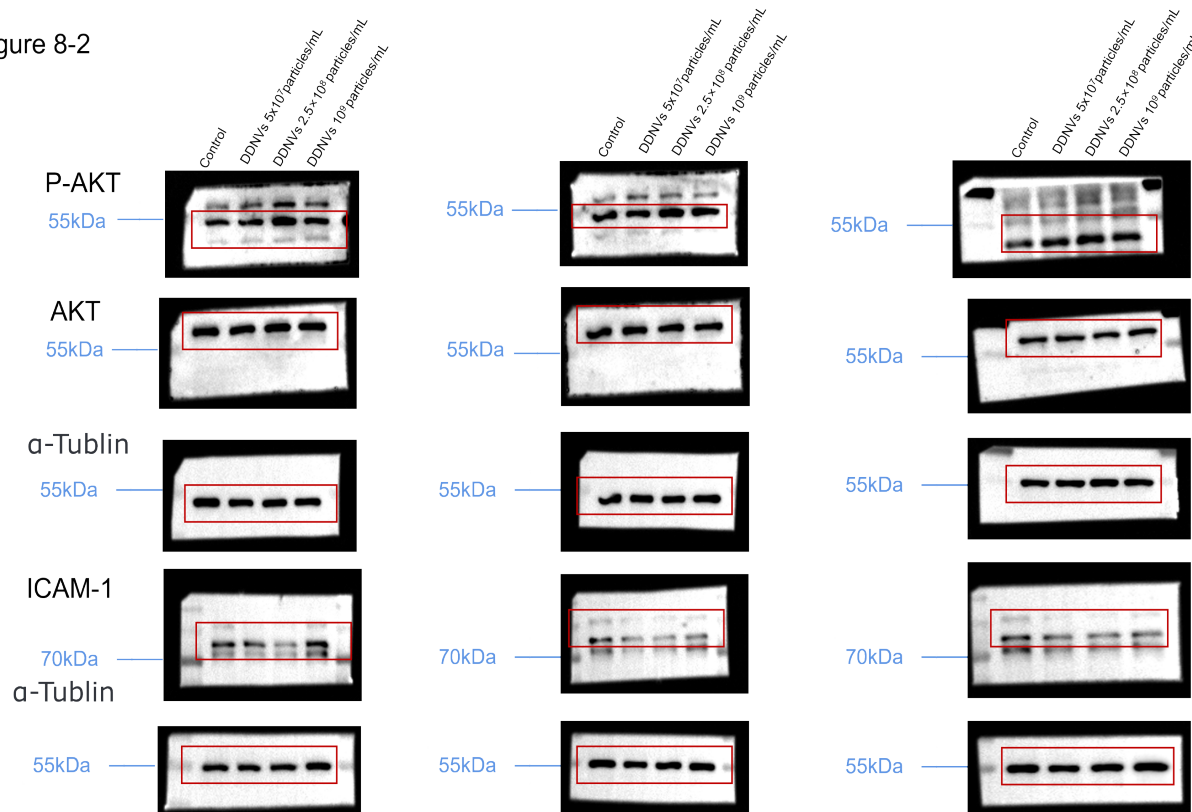

Figure 9-1

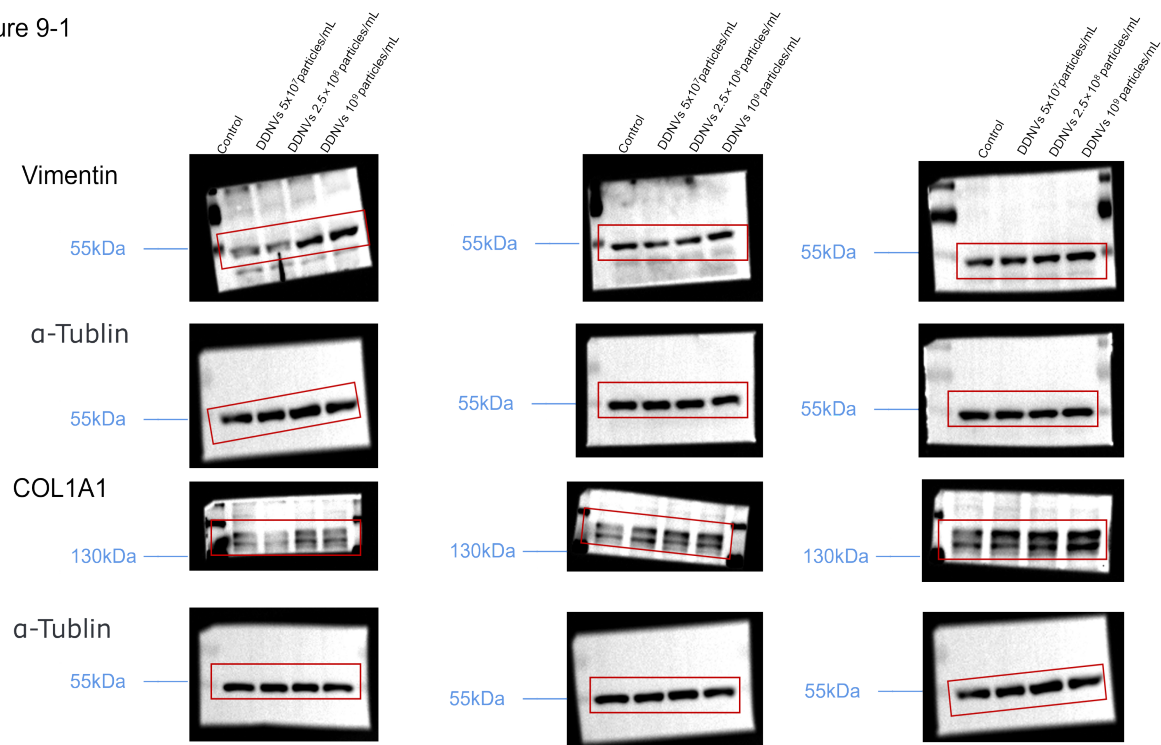

Figure 9-2

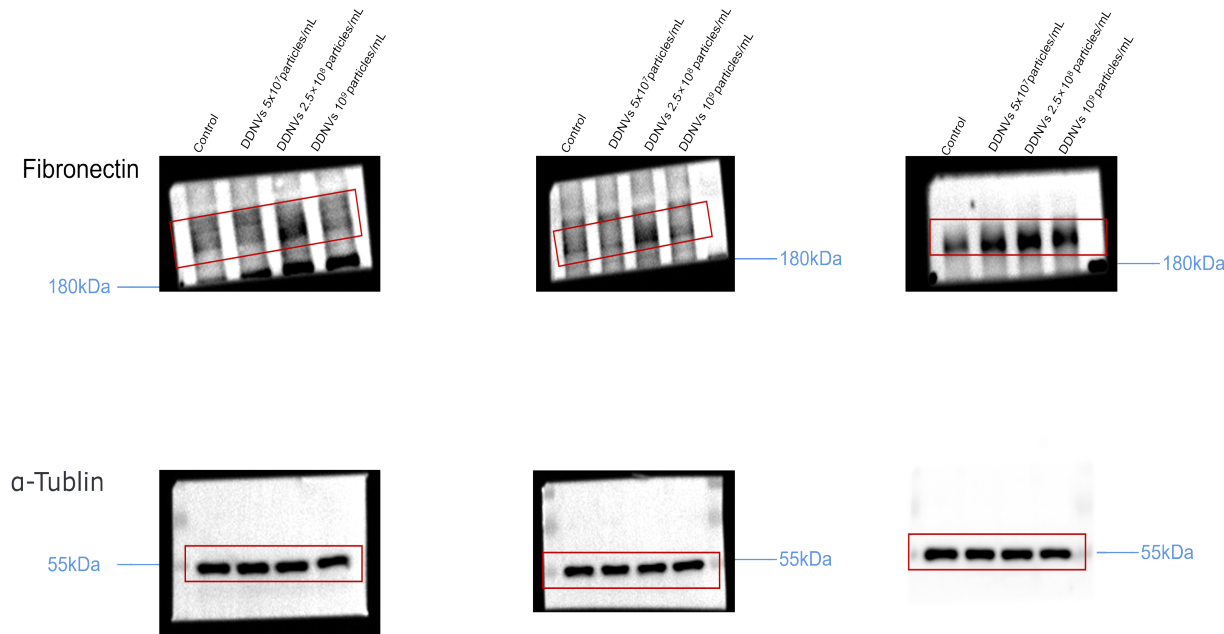

Catalogue numbers of the ladders: Thermo Scientific™ 26616

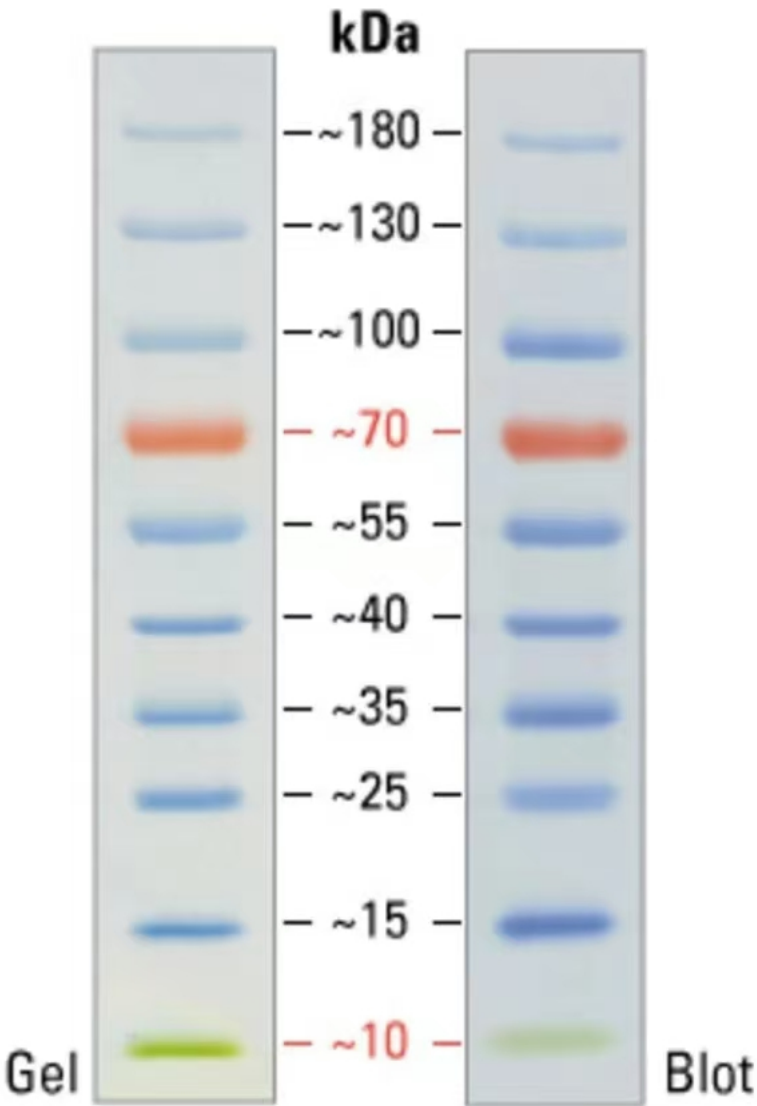

Supplement: Supplementary file 1 — Supplementary Material 1 [file 40643_2025_915_MOESM1_ESM.pdf]
